# RPA-CRISPR/Cas12a assay for the diagnosis of bovine *Anaplasma marginale* infection

**DOI:** 10.1038/s41598-024-58169-6

**Published:** 2024-04-03

**Authors:** Arpaporn Sutipatanasomboon, Jantana Wongsantichon, Somsri Sakdee, Piyaporn Naksith, Amaya Watthanadirek, Panat Anuracpreeda, Stuart D. Blacksell, Chonticha Saisawang

**Affiliations:** 1 Molecular Biosciences Cluster, Institute of Molecular Biosciences, Mahidol University, Salaya Campus, Salaya, Thailand; 2 Mahidol-Oxford Tropical Medicine Research Unit, Faculty of Tropical Medicine, Mahidol University, Bangkok, Thailand; 3 Centre for Tropical Medicine and Global Health, University of Oxford, Oxford, UK; 4 Center for Advanced Therapeutics, Institute of Molecular Biosciences, Mahidol University, Salaya Campus, 25/25 Phuttamonthon 4 Road, Salaya, Nakhon Pathom 73170 Thailand

**Keywords:** Sensors and probes, Assay systems

## Abstract

*Anaplasma marginale* infection is one of the most common tick-borne diseases, causing a substantial loss in the beef and dairy production industries. Once infected, the pathogen remains in the cattle for life, allowing the parasites to spread to healthy animals. Since clinical manifestations of anaplasmosis occur late in the disease, a sensitive, accurate, and affordable pathogen identification is crucial in preventing and controlling the infection. To this end, we developed an RPA-CRISPR/Cas12a assay specific to *A. marginale* infection in bovines targeting the *msp4* gene. Our assay is performed at one moderately high temperature, producing fluorescent signals or positive readout of a lateral flow dipstick, which is as sensitive as conventional PCR-based DNA amplification. This RPA-CRISPR/Cas12a assay can detect as few as 4 copies/μl of *Anaplasma* using *msp4* marker without cross-reactivity to other common bovine pathogens. Lyophilized components of the assay can be stored at room temperature for an extended period, indicating its potential for field diagnosis and low-resource settings of anaplasmosis in bovines.

## Introduction

Anaplasmosis is an infectious blood disease distributed globally across five continents caused by *Anaplasma marginale*, an obligate intracellular parasitic bacterium^1–8^. The parasite is carried by blood-feeding insects such as ticks and flies, and bovines can contract the disease through insect bites or direct contact with traces of blood from infected animals^9^. The disease can be treated with extensive administration of tetracyclines although permanent clearance of the parasites remains questionable^10–12^.

Clinical manifestations of *A. marginale* infection are marked by acute onset fever and other nonspecific symptoms such as decreased appetite and general weakness, similar to other insect-borne infectious blood diseases, such as, babesiosis caused by *Babesia* spp. and trypanosomiasis caused by *Trypanosoma* spp^13–15^. Hence, *A. marginale* infection is often misdiagnosed or diagnosed late in the disease state, and can only be confirmed by the identification of the infectious bacterium.

Conventionally, *A. marginale* infection is determined by microscopic observation, which is inherently subjective, requiring an experienced veterinary pathologist capable of discerning *A. marginale* from other bacterial parasites, and is prone to produce false negatives in cases of low levels of the pathogen^16^. Serological tests such as latex agglutination, complement fixation, indirect fluorescent antibody, and enzyme-linked immunosorbent assay (ELISA) identify the parasite based on the detection of IgG antibodies against *A. marginale* antigens, including major surface protein 2 (MSP2), MSP4, and MSP5^16^. These tests are considered simple, and affordable, providing sufficient accuracy for clinical diagnosis^17,18^; however, cross-reactivity of the chosen antibody can affect the specificity of the test^16,18^.

Molecular detection has been employed in the identification of *A. marginale* in cattle blood samples. Polymerase chain reaction (PCR)-based methods or their modifications have been used to amplify a selected region of the *A. marginale* genome, followed by visualization with gel electrophoresis methods. While identification based on PCR-based DNA amplification is typically more sensitive and specific, it is considered time-consuming and less applicable to fieldwork due to the storage requirement of reagents at low temperatures and specific laboratory instruments such as a thermocycler and high thermal power^16,19,20^. To overcome challenges in point-of-care diagnostics, recombinase polymerase amplification (RPA), a form of isothermal amplification, and Clustered Regularly Interspaced Short Palindromic Repeats (CRISPR)/Cas12a have been combined and applied for the detection without compromising the sensitivity or specificity of molecular detection. In RPA-CRISPR/Cas12a, amplicon specificity is determined by the cleavage of the endonuclease Cas12a, which is guided by a specific CRISPR RNA (crRNA) that recognizes the target sequence^21,22^. The RPA-CRISPR/Cas12 system and the modifications have been successfully developed for one-tube, point-of-care testing of African swine fever, SARS-CoV2, and monkeypox, displaying enhanced sensitivity and accuracy suitable for point-of-care diagnosis^23–27^.

In this study, the RPA-CRISPR/Cas12a system was developed for the diagnosis of *A. marginale* infection in bovines, which, to our knowledge, has not been previously reported. The readouts of our assay can be in fluorescent or colorimetric lateral flow dipstick formats, and the specificity is on par with PCR-based amplification. Also, the test reagents of our assay can be stored at room temperature, making it suitable for on-site diagnosis in low-resource settings, providing an optional on-site diagnostic method that can significantly improve the containment and management of *A. marginale* infection in livestock.

## Results

**Figure 1:**
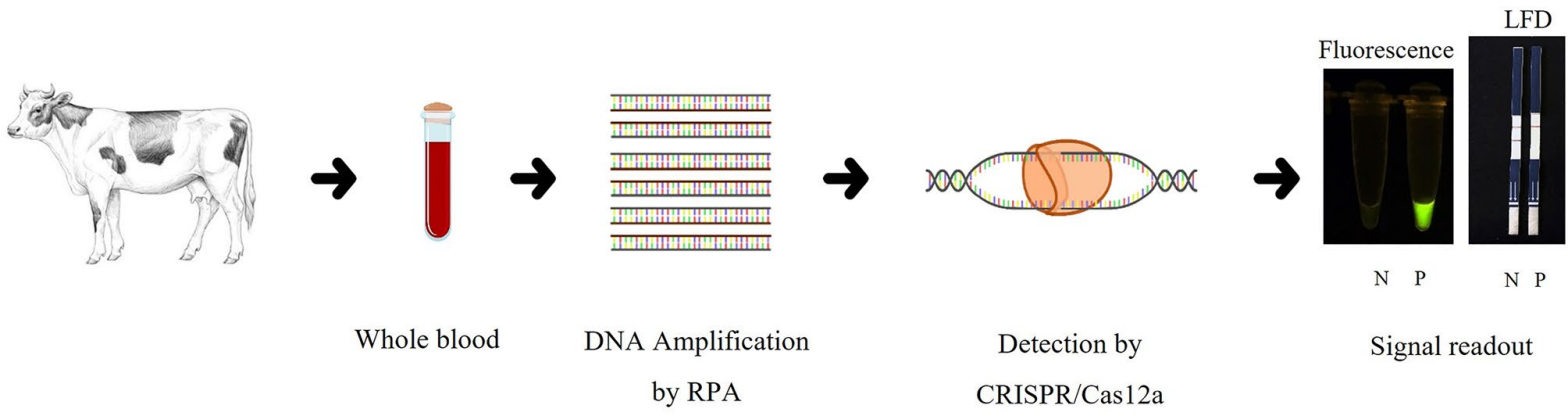
The RPA-CRISPR/Cas12a assay for the diagnosis of *Anaplasma marginale* infection in bovines comprises blood sample collection, genomic DNA extraction, DNA amplification by RPA, and detection by CRISPR/Cas12a. Positive readout (P) can be translated into fluorescent signals or a single-color band in a lateral-flow dipstick (LFD), while negative readout (N) results in the absence of fluorescent signal or two-color bands in LFD.

In our assay, cattle blood samples were collected, and the genomic DNA was extracted for *msp4* amplification by RPA. Following RPA, specific CRISPR RNA (crRNA) was used to guide the Cas12a endonuclease to cleave the RPA amplicons, where positive detection can be translated into fluorescent signals or a positive read-out of lateral-flow dipsticks (LFD) (Fig. 1).

### Development of the RPA-CRISPR/Cas12a assay

**Figure 2:**
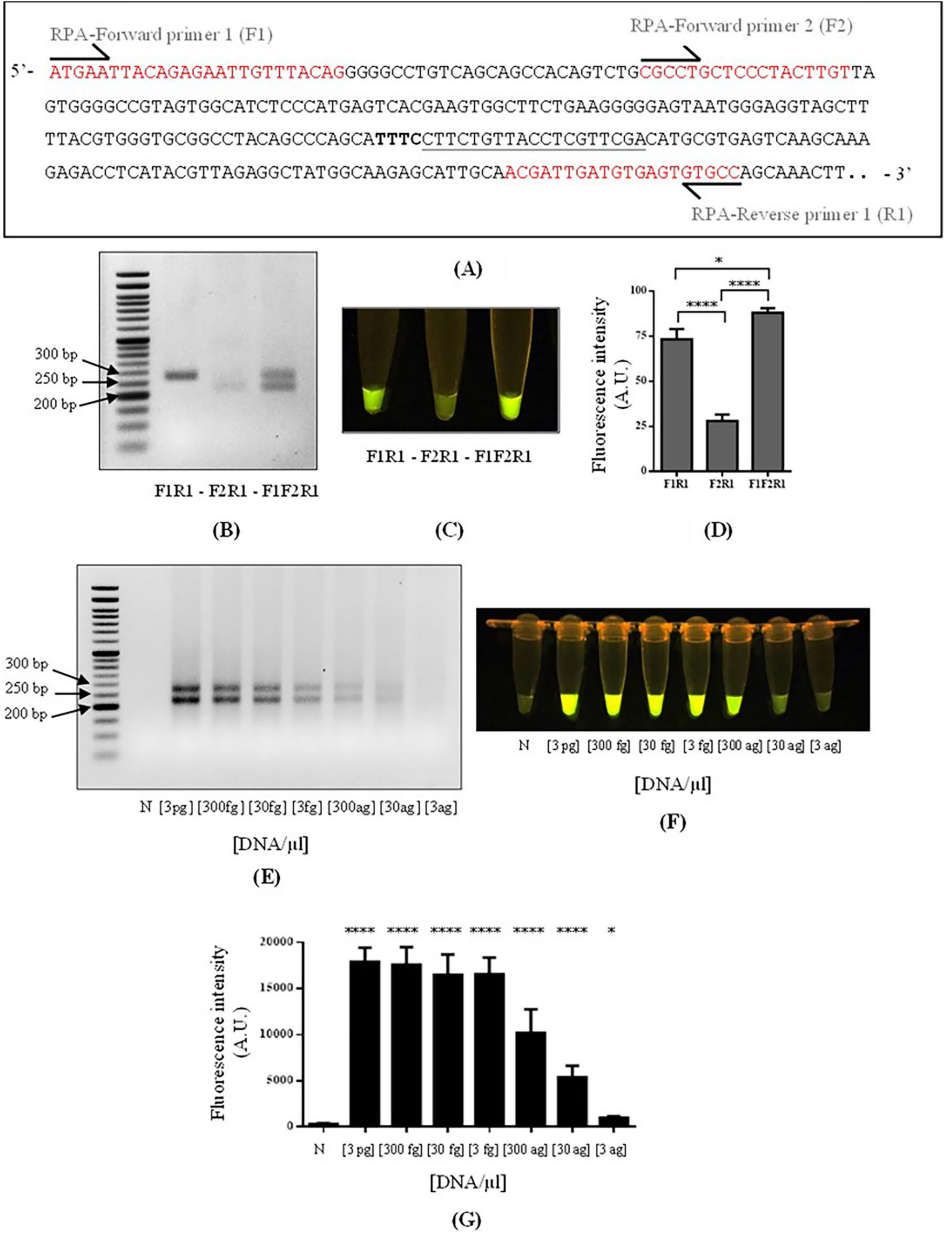
Validation of RPA-CRISPR/Cas12a assay detection. (**A**) The target sequence of the *A. marginale msp4* gene region. RPA primers consist of 2 forward (F1 and F2) and 1 reverse (R1) direction as shown in red were designed to flank the PAM sequence (bold letters) and the crRNA sequence (underlined). (**B**–**D**) Primer combination for assay condition optimization. The agarose gel of RPA products and corresponding fluorescent signals of CRISPR/Cas12a detection were shown, respectively. The bar graph represents the data of mean ± SD from 3 independent experiments. One-way ANOVA with a Tukey's multiple comparisons test. ∗*p* < 0.05, ∗∗*p* < 0.01, ∗∗∗*p* < 0.001, ∗∗∗∗*p* < 0.0001. (**E**) Limit of detection (LOD) test**.** Gel electrophoresis showing RPA amplification products of serially diluted *msp4* recombinant plasmid. The starting concentration of the plasmid template is indicated below each lane. N is no template control. L is 50 bp DNA ladder (ExcelBand™). (**F**,**G**) Fluorescent signals of CRISPR/Cas12a reactions from 1 μl of RPA products in (**E**). The fluorescence intensities were quantitated by ImageJ and plotted by GraphPad Prism version 6 (GraphPad software, Boston, USA). The data represents as mean ± SD from 9 independent experiments. One-way ANOVA with a Tukey's multiple comparisons test. ∗*p* < 0.05, ∗∗*p* < 0.01, ∗∗∗*p* < 0.001, ∗∗∗∗*p* < 0.0001 versus control.

RPA using F1-R1 or F2-R1 as primers resulted in 269 bp and 219 bp amplified fragments, respectively, and the combination of all three primers (F1-F2-R1) produced both of the amplified fragments (Fig. 2B). The RPA products from all primer combinations were subsequently detected by CRISPR/Cas12a using an equal molar amount of the crRNA. The three-primer combination displayed the most intense fluorescence, followed by F1-R1 and F2-R1 combinations, respectively (Fig. 2C,D). For this reason, the three-primer combination was chosen for our RPA-CRISPR/Cas12a assay.

### Limit of detection (LOD)

To determine the limit of detection of our *Anaplasma* diagnosis by RPA-CRISPR/Cas12a fluorescent assay, *msp*4 plasmid was used as a DNA template in a tenfold serial dilution covering a range from 3 pg to 3 ag. Only one-fifth of RPA amplification product was subsequently applied to CRISPR/Cas12a assay and the rest was loaded onto agarose gel electrophoresis to assess amplification efficiency (Fig. 2E). Fluorescent readout of the end product from RPA-CRISPR/Cas12 assay demonstrates the assay could distinctly detect as low as 30 ag/μl of the starting DNA material (Fig. 2F,G), equivalent to 4 copies/μl of *Anaplasma msp4* as calculated from the size of plasmid DNA template. LOD for this assay, in comparison to the PCR method, was additionally investigated by applying % hit rate plots to determine copies of starting DNA material required at 95% confidence^27^. The same set of primers were used for both assays. However, only one 219-bp DNA product band was visually observed by PCR method despite intensive optimizations. At 95% confidence, data suggest the two assays have equivalent LOD of 21 and 20 copies/µl for RPA and PCR amplification, respectively (Supplementary Table 1, Supplementary Fig. 1).

### Cross-reactivity test

**Figure 3:**
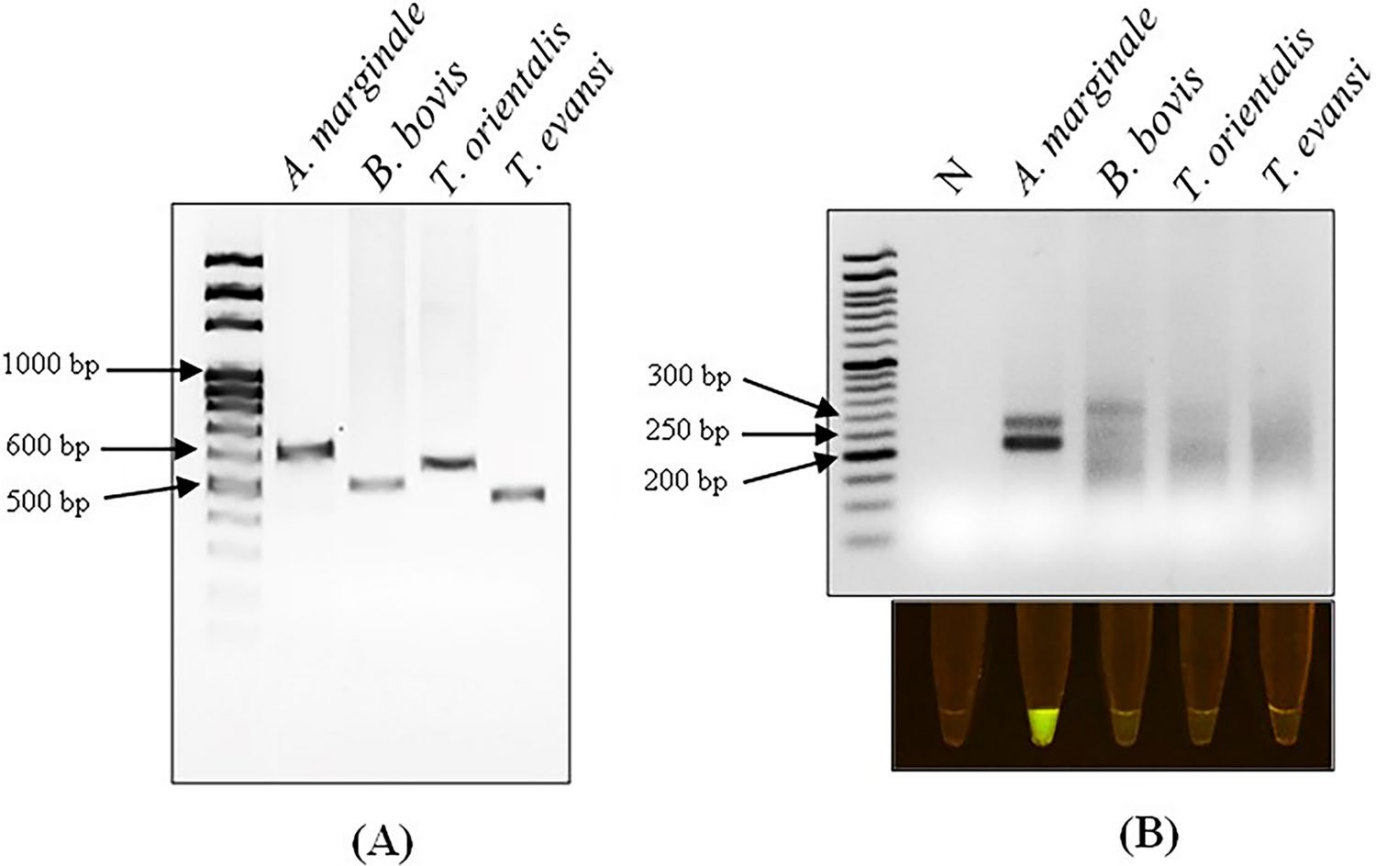
Cross-reactivity evaluation. (**A**) Gel electrophoresis showing PCR amplification of genomic DNA extracted from cattle blood infected with *A. marginale*, *B. bovis*, *T. orientalis*, or *T. evansi* (L: 100 bp DNA ladder (Solis BioDyne, Estonia). The target genes and their primer sequences are listed in Table 1. (**B**) The agarose gel image of RPA reaction and the fluorescent signals of CRISPR/Cas12a reactions using genomic DNA extracted from cattle blood infected with *A. marginale*, *B. bovis*, *T. orientalis*, or *T. evansi* (N: no template control). (L: 50 bp DNA ladder (ExcelBand™).

To evaluate the detection of *A. marginale* infection and the extent of cross-reactivity, we challenged the assay with genomic DNA extracted from blood samples of bovines infected with other vector-borne diseases. The infecting pathogen was confirmed by PCR-based DNA amplification targeting the *msp5* gene of *A. marginale*, *sbp2* (spherical body protein 2) of *B. bovis, p23* of *T. orientalis,* and the *18S* region of *T. evansi* (Fig. 3A) before the samples were used as a template for the RPA-CRISPR/Cas12a assay. As a result, only the samples with confirmed *A. marginale* infection displayed visible fluorescent signals, establishing that our RPA-CRISPR/Cas12a assay is specific to *A. marginale* infection (Fig. 3B).

### Assay performance

The accuracy of our RPA-CRISPR/Cas12a was evaluated using the genomic DNA extracted from 100 cattle blood samples. PCR-based DNA amplification targeting the *msp4* gene established that half of the blood samples were confirmed positive and half were negative for *A. marginale* infection (Supplementary Fig. 2). When using the same amount of DNA template (3 ng) in our assay, all 50 samples that were confirmed negative by PCR tested negative, 46 out of 50 samples that were confirmed positive by PCR tested positive, and 4 out of 50 produced false negatives (Supplementary Table 2, Supplementary Fig. 3). Accordingly, the assay's sensitivity, specificity, and accuracy were 92, 100, and 96%, respectively (MedCalc Software Ltd. Diagnostic test evaluation calculator; Version 22.009).

### Stability and storability test

**Figure 4:**
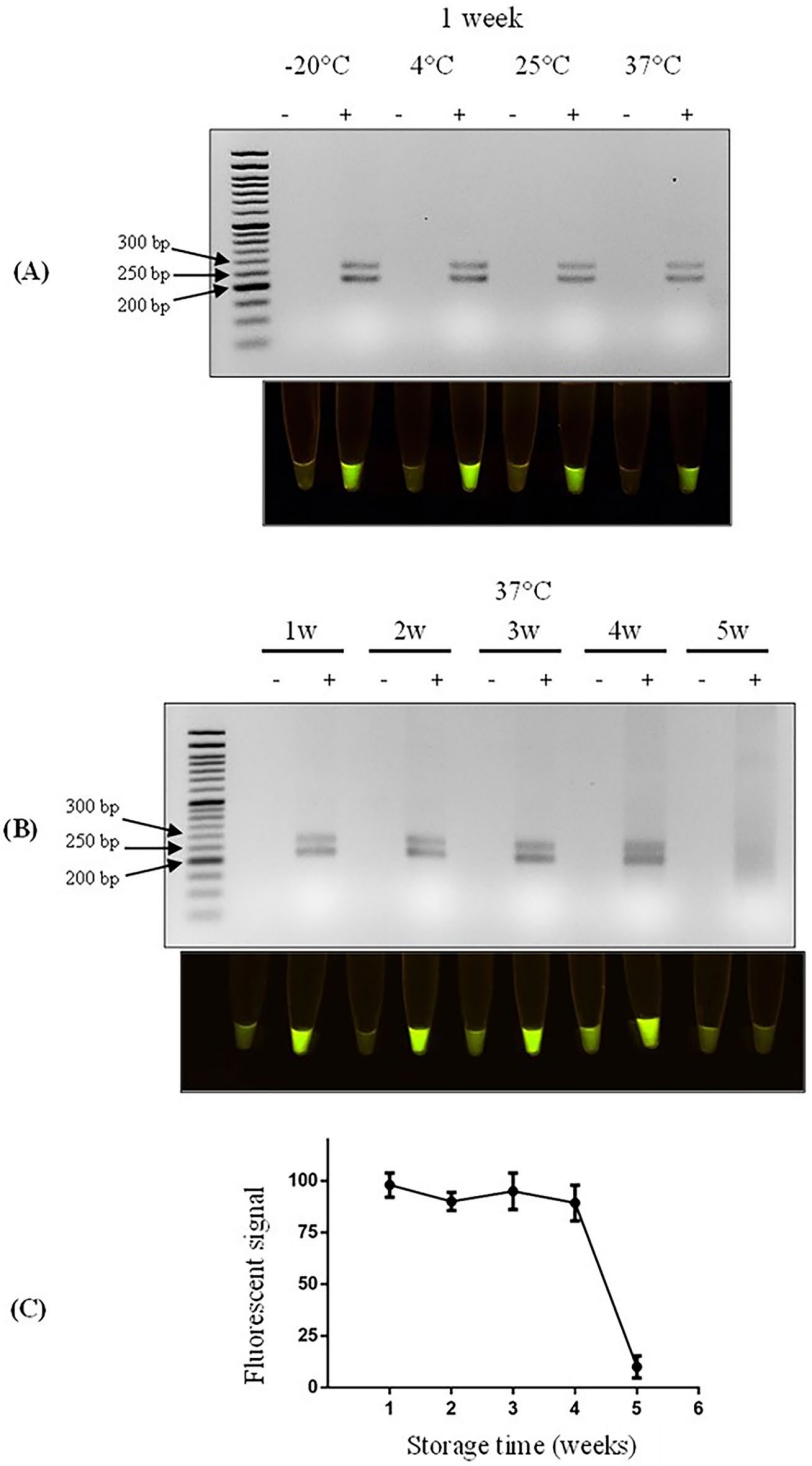
Stability and storability test. (**A**) The RPA-CRISPR/Cas12a results from resuspended lyophilized RPA and CRISPR/Cas12a components, which were stored at different temperatures for 1 week. (**B**,**C**) The RPA-CRISPR/Cas12a results from resuspended lyophilized RPA and CRISPR/Cas12a components, which were stored at 37 °C from 1 week up to 5 weeks. The average intensities of the fluorescent signals were quantitated and represented as mean ± SD. n = 3.

**Figure 5:**
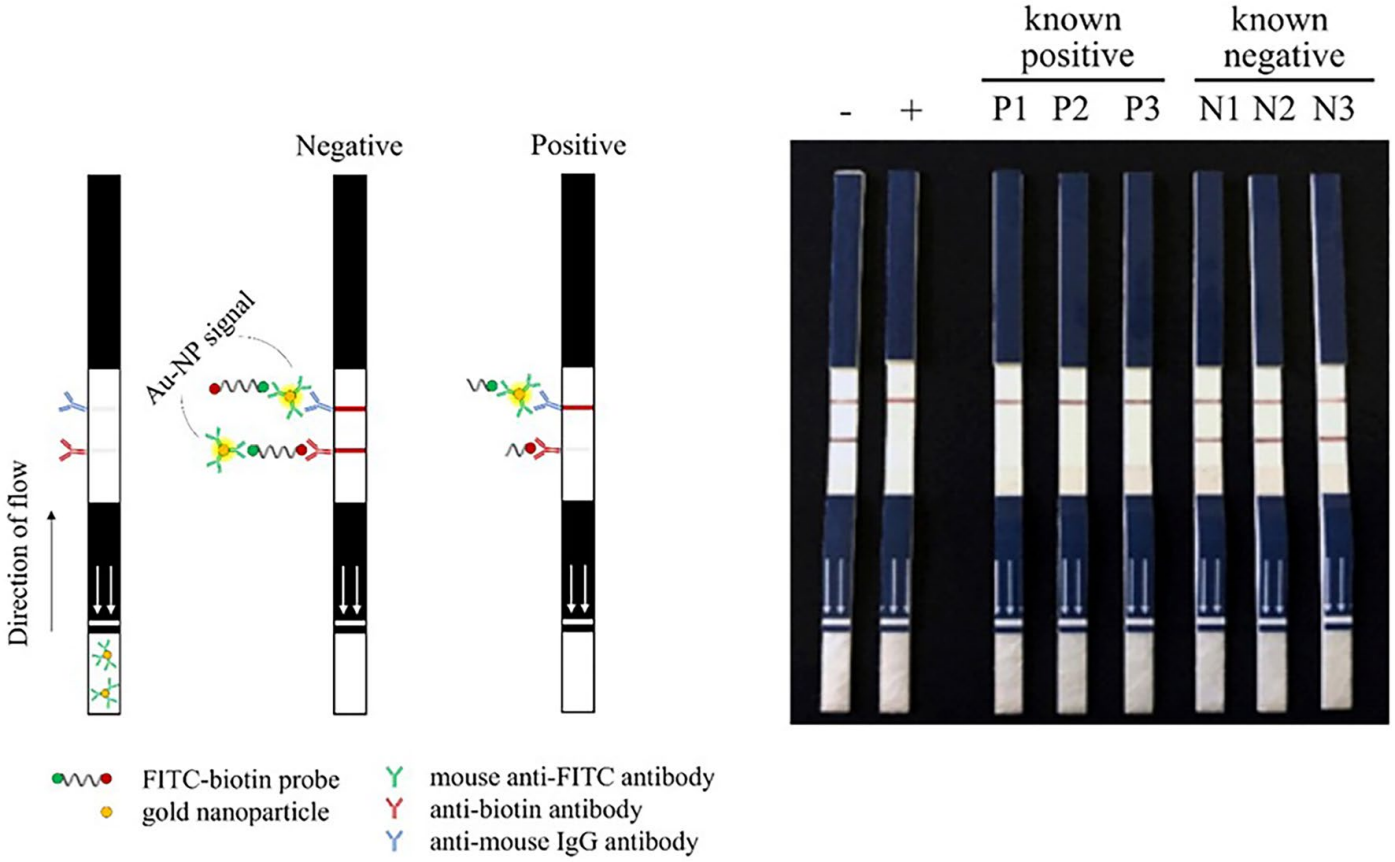
Schematic representation of the lateral flow dipstick chromatography (LFD) and visual readouts of RPA-CRISPR/Cas12a reaction. The white arrows on the dipsticks indicate the end of the stick to dip. The following templates are shown: (−) plasmid DNA, (+) *msp4* recombinant plasmid DNA, (P1–P3) genomic DNA extracted from blood drawn from bovines known to be infected with *A. marginale*, and (N1–N3) genomic DNA extracted from blood drawn from bovines free from *A. marginale* infection.

Resuspended lyophilized reactions produced true negative and positive results regardless of the storage temperature (Fig. 4A). The fluorescent signals from lyophilized reactions stored at 25 and 37 °C were lower than those stored at − 20 or 4 °C. Nonetheless, the fluorescence emanating from positive samples is distinct from negative samples so that it can be correctly interpreted, indicating that the assay is stable and stored at room temperature. Next, we extended the storage time at 37 °C from 1 week to up to 5 weeks. At the first four time points, the resuspended lyophilized reactions could produce true negatives and positives, suggesting its capability for long-term storage (Fig. 5B,C).

### Lateral flow dipsticks

As fluorescent signal detection requires a specialized transilluminator, we adapted the assay readout to lateral-flow dipsticks (LFD). The RPA-CRISPR/Cas12a reaction solutions were applied at the conjugate pad of the LFD. For a negative sample, where the fluorescein (FITC)-biotin probes are not cleaved by Cas12a enzyme, the intact probes will interact with the gold nanoparticle (AuNP) at the conjugate pad and then bind to the anti-BIOTIN capture antibody. An excess solution containing an uncleaved probe will travel forward and bind to an anti-mouse IgG antibody, resulting in the appearance of two bands on a strip. In contrast, the positive sample appears as a single band as the probes have been cleaved. The FITC side of the probe will bind to the anti-FITC capture antibody embedded on the AuNP and thereby be trapped by the anti-mouse IgG antibody (Fig. 5). The presence of AuNP generates the signal through surface plasmon resonance of the gold nanoparticle, which is observed as a red fluorescence. The cleaved probe containing the biotin will still bind to the anti-biotin antibody, however the AuNP is lost after cleavage and no signal will be observed. The resulting reaction of *msp4* recombinant plasmid and genomic DNA extracted from samples positive for *A. marginale* infection appeared as one colored band, whereas genomic DNA obtained from samples negative for *A. marginale* infection resulted in two colored bands (Fig. 5). Thus, LFD can be used as the readout of our RPA-CRISPR/Cas12a assay instead of fluorescent signals.

## Discussion

The uncertainty in diagnosis of the disease also has major public health concerns and economic impact on farmers. In Thailand’s rural areas, several tick-borne diseases exist^28^. Livestock husbandry, especially cattle, are fed by wandering and grazing natural bush in open areas where sanitation is poorly controlled. Thus, cattle are at high risk of infection, resulting in substantial losses to rural revenue^29^. Various epidemiological surveys of tick-borne pathogen species have been performed in different parts of Thailand. The infections were confirmed by PCR molecular detection methods largely focusing on the *msp* (major surface protein) gene for Anaplasmataceae family^3,30^. Over 65% of infection rates were identified as *A. marginale* from positive blood samples collected from 18 provinces over 5 different regions of Thailand^31^. However, the prevalence of the disease varies due to different seasons and times, even though the blood samples were collected from the same region of the country^32,33^.

Several diagnostic methods for *A. marginale* have been well established^34^. However, the optimum diagnosis depends on the detection time relative to the time of infection and species identifiability from testing. This study reports a new nucleic acid detection method for *A. marginale* pathogen in infected cattle. The major surface protein 4 gene (*msp4*) was selected as a gene target. MSP4 is one of six members that belong to the major surface protein (MSP) superfamily. These proteins play a crucial role in the adhesion of *A. marginale* to host cells’ erythrocytes causing the infection^11^. MSPs are thus the most likely gene candidates selected for *Anaplasma* spp. for molecular detection. Particularly *msp4* and *msp5* that are shown to be highly conserved because they have originated from single genes^35,36^. Besides *msp*, other genes such as *ankA, groESL* and 16S rRNA, are occasionally selected as target genes^37^. The 16S rRNA gene is also widely used as a marker for bacterial identification. Nonetheless, the efficiency of detection would also depend on the gene regions that are amplified. Some areas of the gene are more conserved compared to other regions of the same gene^38^. Our detection assay comprises two steps after genomic DNA preparation: specific gene amplification and signal detection. For the first step, the gene target is amplified by isothermal nucleic acid amplification, recombinase polymerase amplification (RPA)^39^. RPA has revolutionized the isothermal amplification technology and has been used to detect a wide range of pathogens in human and veterinary medicine, agriculture, and food safety^40–43^. Similar to conventional PCR, a pair of primers is needed to amplify the target spanning the amplicons that are not longer than 300 bp for the most efficient amplifiability. To obtain more amplicon products, we employed two forward primers (F1 and F2) to pair with one reverse primer (R1), yielding two amplified fragments. This strategy has drastically increased the amplified products and the assay's sensitivity for the final detection. Both amplified products contain the protospacer adjacent motif (PAM) required for the CRISPR/Cas12a detection system in the second step. PAM sequence is a short DNA motif required for the Cas endonuclease enzyme to recognize and then cleave on the DNA target. Initially, the Cas12a enzyme forms a complex with CRISPR RNA (crRNA), guides RNA for sequence-specific targets, and then starts searching for the PAM recognition sequence (TTTV). Once the PAM sequence is found, the crRNA binds to the complementary sequence of the target DNA adjacent to the PAM sequence. The enzyme now undergoes major conformational changes that activate catalytic activity, followed by cleavage of the double-stranded target DNA. After cleavage, the Cas12a enzyme remains activated, exhibiting a collateral cleavage activity where the enzyme can cleave the surrounding single-stranded DNA (ssDNA) non-specifically^44–46^. CRISPR/Cas12a detection assay contains a reporter probe consisting of a quencher and a fluorophore with a short ssDNA. Consequently, the activated Cas12a enzyme collaterally cleaves the nearby reporter probe at the ssDNA, separating the quencher and fluorophore, allowing the fluorescence to be detected^47^. The fluorescence intensity represents the amount of DNA that has been amplified, which correlates with the ability of the crRNA to bind to the target DNA in a complementary manner. The performance of the assay is determined by a limit of detection (LOD). This study demonstrated that the RPA-CRISPR/Cas12a assay targeting *msp4* for *A. marginale* detection is excellent. We can detect the starting DNA material down to 30 ag, which is equal to 4 copies/μl of *msp4* per reaction. The clinical validation was also performed with cattle blood samples. The diagnostic performance in sensitivity, specificity, and accuracy are met. A cross-reaction with other pathogens causing similar clinical symptoms to anaplasmosis was not observed. The fluorescent signal of the detection can be visualized by a portable device. The lateral flow assay format can also be conveniently deployed by changing reporter probes labeled with reporter molecules corresponding with the antibodies bound to the strip.

Initially, both of these detection formats offer a simplified approach to nucleic acid detection with some technical limitations. This was due to several components of the assay reaction, such as fluorescent probes and enzymes, requiring an ultra-low temperature to protect them from degradation and denaturation. Therefore, employing this detection kit would be difficult at a point of care, especially in rural areas where the cold chain transportation and electricity could not be accessed. Several studies have attempted to prolong the stability of the reaction components at ambient temperature^48–50^. In this study, we have overcome this issue by adding specific stabilizers to preserve the structure of the biological molecules. The lyophilized reactions remain stable for up to a month, even stored at high temperatures (37 °C), thereby maintaining the activity of the protected components. Moreover, the assay operation has been simplified by putting all reaction components in one tube for each reaction step, resulting in a highly convenient detection kit for the end user.

## Conclusion

The RPA-CRISPR/Cas12a assay developed in this study provides an alternative to identifying *A. marginale* by DNA amplification. The specificity and accuracy of our assay are on par with conventional PCR-based assays. The assay can be performed in under an hour at a moderately high temperature (39 °C), achievable with a conventional incubator, heat block, or lukewarm water bath. The lyophilization of the assay components and readout by LFD also demonstrate its potential for long-term storage and on-site field diagnosis of anaplasmosis in bovines.

## Materials and methods

### Study areas and sample collection

The cattle blood samples in this work were obtained from previous studies^32,51^. They are collected from different areas in the northern and northeastern parts of Thailand. Briefly, blood samples were collected from the jugular vein of cattle, which were chosen randomly regardless of their clinical symptoms. Fifty *A. maginale*-infected and non-infected samples were selected for this study. All study protocols were approved by the institutional committee for the use and care of laboratory animals (Ethical Approval Protocol No. IMB-ACUC 2023/008) and were performed in accordance with the relevant guidelines and regulations. These studies were also reported as described by the ARRIVE guidelines 2.0. Once collected, the sample is transferred to an individual tube containing ethylenediaminetetraacetic acid (EDTA) buffer solution and kept at 4 °C.

### Nucleic acid preparation

A recombinant plasmid containing the *A. marginale msp4* gene obtained from previous study^36^ was used as a DNA template for positive controls in PCR and RPA. The recombinant plasmid was transformed and maintained in *Escherichia coli* DH5α, cultured overnight, and the plasmid was extracted using the Large Plasmid DNA Extraction Kit (Geneaid Biotech Ltd.). The plasmid was verified based on the insert size using restriction digestion and agarose gel electrophoresis.

Genomic DNA was extracted from 200 μl of whole blood by a High Pure PCR Template Preparation kit (Roche) following the manufacturer's protocol and kept at − 20 °C until used.

### Examination of parasitic infections and PCR-based DNA amplification

Collected blood samples were analyzed for infection with *A. marginale*, *Babesia bovis*, *Theileria orientalis*, and *Trypanosoma evansi* by PCR-based DNA amplification using the extracted genomic DNA. Each PCR reaction contained 1 μM of forward and reverse primers, 200 μM of each dNTP, 1× Phusion™ HF buffer, 0.5 U Phusion DNA polymerase (Thermo Scientific™, F-530S), and 50 ng of the template DNA unless indicated otherwise (Supplementary Table 3). PCR reactions were performed using the following condition: 94 °C for 5 min, 30 cycles of 94 °C for 1 min, 55 °C for 1 min, 68 °C for 1 min, and 68 °C for 7 min. The PCR products were separated by 1% agarose gel electrophoresis and stained with 0.1% ethidium bromide solution. The target gene for each pathogen and the sequence of forward and reverse primers, including the expected length of the PCR amplicon(s), are listed in Table 1.

**Table 1:** Target genes and primer sequences for the detection and identification of the infectious agents and the corresponding amplicon lengths.

Target	Primer name	Amplicon length (bp)	Sequence (5'–3')
*Anaplasma marginale* (*msp4*)	RPA-forward primer F1	269	ATGAATTACAGAGAATTGTTTACAG
	RPA-forward primer F2	219	CGCCTGCTCCCTACTTGT
	RPA-reverse primer R1	/	GGCACACTCACATCAATCGT
CRISPR	crRNA	/	CTTCTGTTACCTCGTTCGA
*Anaplasma marginale* (*msp4*)	*msp4*-forward	849	ATGAATTACAGAGAATTGTTTACAGG
	*msp4*-reverse		TTAGCTGAACAGGAATCTTGCTCC
*Anaplasma marginale* (*msp5*)	*msp5*-forward	633	CACCATGAGAATTTTCAAGATTGTGTC
	*msp5*-reverse		CTAAGAATTAAGCAT
*Babesia bovis (sbp2)*	*sbp*-forward	584	CGAATCTAGGCATATAAGGCAT
	*sbp*-reverse		ATCCCCTCCTAAGGTTGGCTAC
*Theileria orientalis* (*p23*)	*p23*-forward	601	GTACACACCTTGAAATCTGGC
	*p23*-reverse		CAAGAGAGGCAACAACAACGA
*Trypanosoma evansi* (*18S*)	*18S*-forward	538	CGTCCCTGCCATTTGTACACAC
	*18S*-reverse		GGAAGCCAAGTCATCCATCG

### DNA amplification by recombinase polymerase amplification (RPA)

For the DNA amplification step, three primers were designed to target the *msp4* gene of *A. marginale*. The combination of the reverse primer R1 and the forward primer F1 or F2 flanks the nucleotides of the protospacer adjacent motif (PAM) sequence (5ʹ-TTTC-3ʹ) and the crRNA sequence (5ʹ-CTTCTGTTACCTCGTTCGA-3ʹ), which are required for the downstream CRISPR/Cas12a detection (Fig. 2A).

DNA amplification by RPA was performed using TwistAmp™ Basic Kit (TwistDX). Either recombinant DNA or genomic DNA was used as a template. Briefly, 0.3 μM of each forward primer, 0.6 μM of the reverse primer targeting the *A. marginale msp4* gene were added to 29.5 μl of rehydration buffer to resuspend lyophilized components, and 14 mM of magnesium acetate was added just before the reaction commenced to a final volume of 50 μl (Supplementary Table 3). RPA was performed at 39 °C for 15–30 min, and the amplification was stopped by incubating at 75 °C for 5 min. The RPA products were preliminarily verified by 2% (w/v) agarose gel electrophoresis before detection by CRISPR/Cas12a. The sequence of forward and reverse primers, including the expected length of the RPA amplicon, are listed in Table 1.

### Cas12a expression and purification

The Cas12a protein was expressed and purified from *E. coli* BL21, harboring the Cas12a plasmid (Addgene plasmid #114366). The expression, purification, and quantification of Cas12a were performed as previously described^52^. The purity of Cas12a was visualized on 10% SDS-PAGE gel, stored in 50% glycerol, and kept at − 20 °C until used.

### CRISPR/Cas12a detection

The RPA products were detected in a CRISPR/Cas12a reaction containing 1 μl of RPA product, 30 nM crRNA, 1X NEBuffer 2.0, 50 nM Cas12a enzyme, and 1.5 μM of the fluorescent reporter of single strand DNA probe with fluorescein (FAM) and black hole quencher 1 (BHQ1) at the 5ʹ and 3ʹ ends, respectively (Supplementary Table 3). The reaction was incubated at 39 °C for 15–30 min, and the resulting fluorescence was visualized by eyes using BluPAD Dual LED Blue/White Light Transilluminator (Bio-helix, BP001CU). For detection by lateral flow dipsticks (LFD) with the use of Amplicon detection 1T1C dip strip (Serve Science Co. Ltd., Thailand), the reaction was performed in the same manner but with a fluorescein (FITC)-biotin probe instead of FAM-quencher probe.

### Limit of detection (LOD)

The developed assay's limit of detection (LOD) was evaluated using the *msp4* recombinant plasmid. The starting concentration of the DNA template was determined using the NanoDrop 2000™ spectrophotometer (Thermo Scientific), and 3 pg/μl of the plasmid DNA was serially diluted tenfold until the final concentration reached 3 ag/μl. For RPA-CRISPR/Cas12a, the images were used to quantify the fluorescence intensity by ImageJ^53^ and the values from independent RPA-CRISPR/Cas12a experiments were averaged, analyzed, and plotted by GraphPad Prism version 6 (GraphPad software, Boston, USA.).

### Storage and stability of assay components

The assay components were tested for storage stability after freeze-drying. To increase the stability and storability of the assay, we used a combination of cryoprotectants and freeze-dried all components to preserve the enzymes and crRNA used in the assay. Cryoprotectants were added to RPA and CRISPR/Cas12a components before lyophilization. For RPA, 10% trehalose, 5% polyethylene glycol 35 (PEG35), and 0.1% Triton X-100 were used, and 10% trehalose, 5% pullulan, and 0.1% Triton X-100 were for CRISPR/Cas12a. Lyophilization was performed separately using a Flexi-Dry™ MP freeze-dryer (Kinetics, USA) for 1 h or until the reactions were wholly dried, as previously described ^52^. To test the storage stability of assay components at different temperatures, the lyophilized reactions were stored at − 20, 4, 25, or 37 °C for 1 week and tested with genomic DNA extracted from blood samples that tested positive for *A. marginale* infection. The stability of freeze-dried components at 37 °C were assessed by storing the lyophilized reagents at 37 °C for 1, 2, 3, and 4 weeks. After each designated storage time, the lyophilized reagents were kept at − 30 °C until all reactions could be resuspended and tested simultaneously.

### Ethical approval and consent to participate

Blood sample collection protocol, including sampling number and the handling of animals, was approved by the Institutional Animal Care and Use Committee (IACUC), Institute of Molecular Biosciences, Mahidol University (Protocol No. IMB-ACUC 2023/008), with consent to collect blood samples from owners at the collection site.

### Supplementary Information

Supplementary Information 1.

Supplementary Information 2.

## Data Availability

All data presented in this study are available in the article.
