# Supplementary material for: RPA-CRISPR/Cas12a assay for the diagnosis of bovine Anaplasma marginale infection
Source: Sci Rep. 2024 Apr 3;14:7820. doi: 10.1038/s41598-024-58169-6 (PMC10991388; doi:10.1038/s41598-024-58169-6)
Supplement: Supplementary file 1 — Supplementary Information 1. [file 41598_2024_58169_MOESM1_ESM.pdf]

Supplementary information

1. Supplementary figure

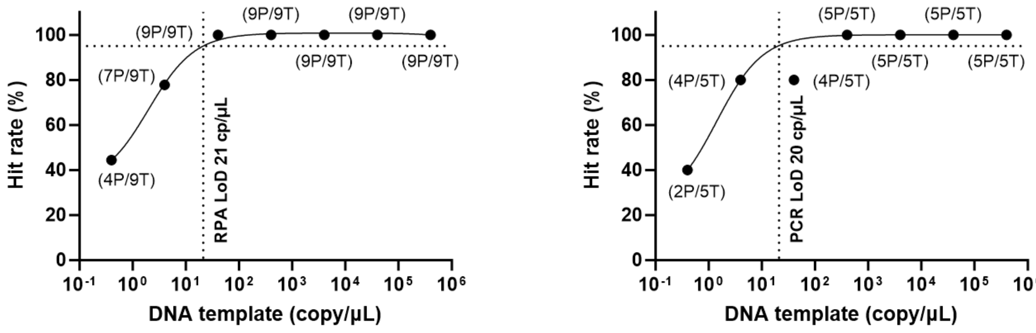

(A) (B)

Supplementary Fig. 1:

(A) LOD of RPA reaction (B) LOD of PCR reaction. Both RPA and PCR reaction employed the same set of primers for amplification (F1F2R1). The concentration of the plasmid template is indicated in **Supplementary Table 1 (A-D)**. Hit Rate plots represent the LOD of reactions at a confidence level of 95%. (P = positive, T = total reactions)

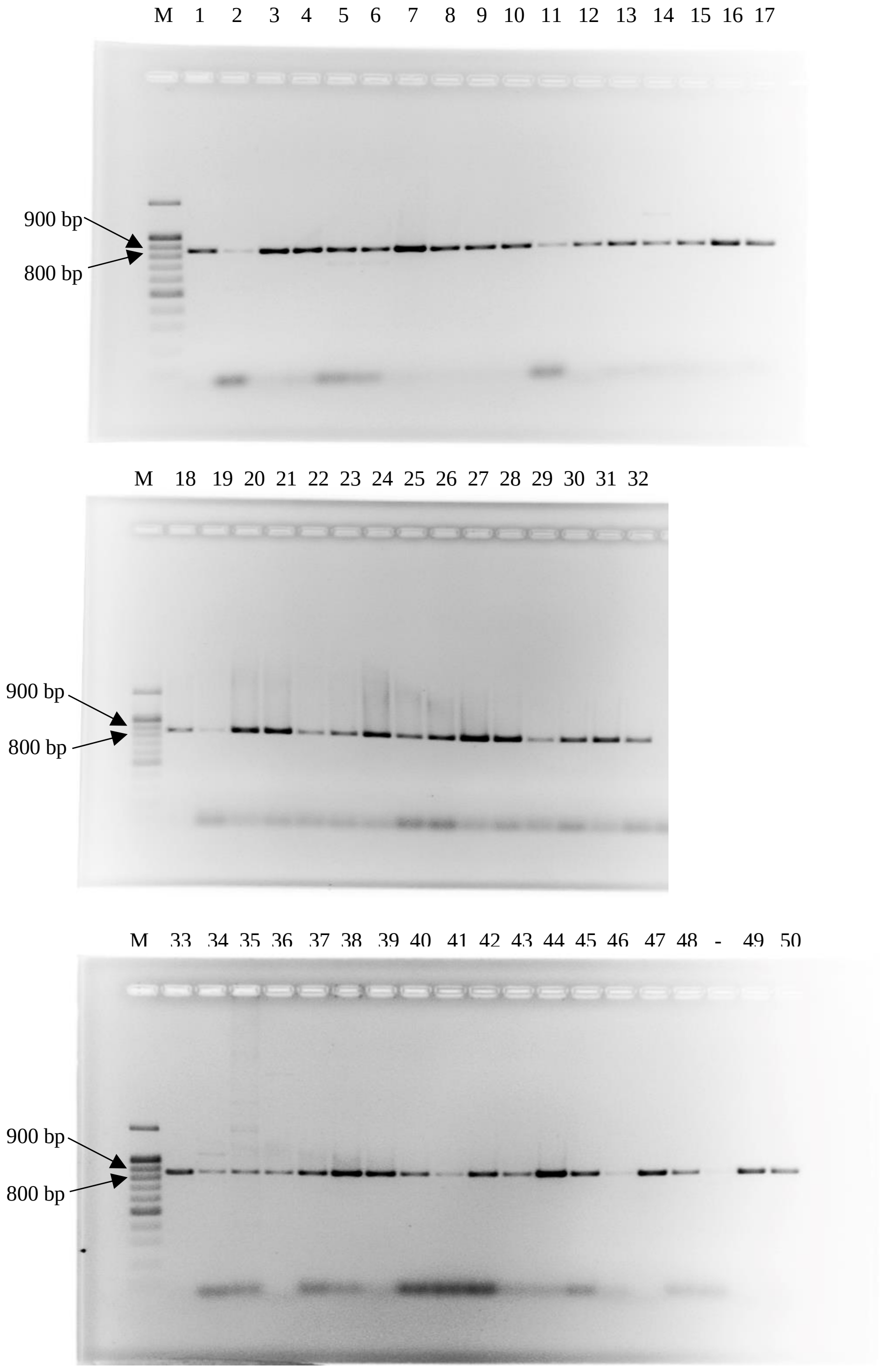

Supplementary Fig. 2: Uncropped agarose gel images showing PCR amplification of genomic DNA extracted from blood samples of bovines infected with *A. marginale*. *Msp4* is 849 bp.

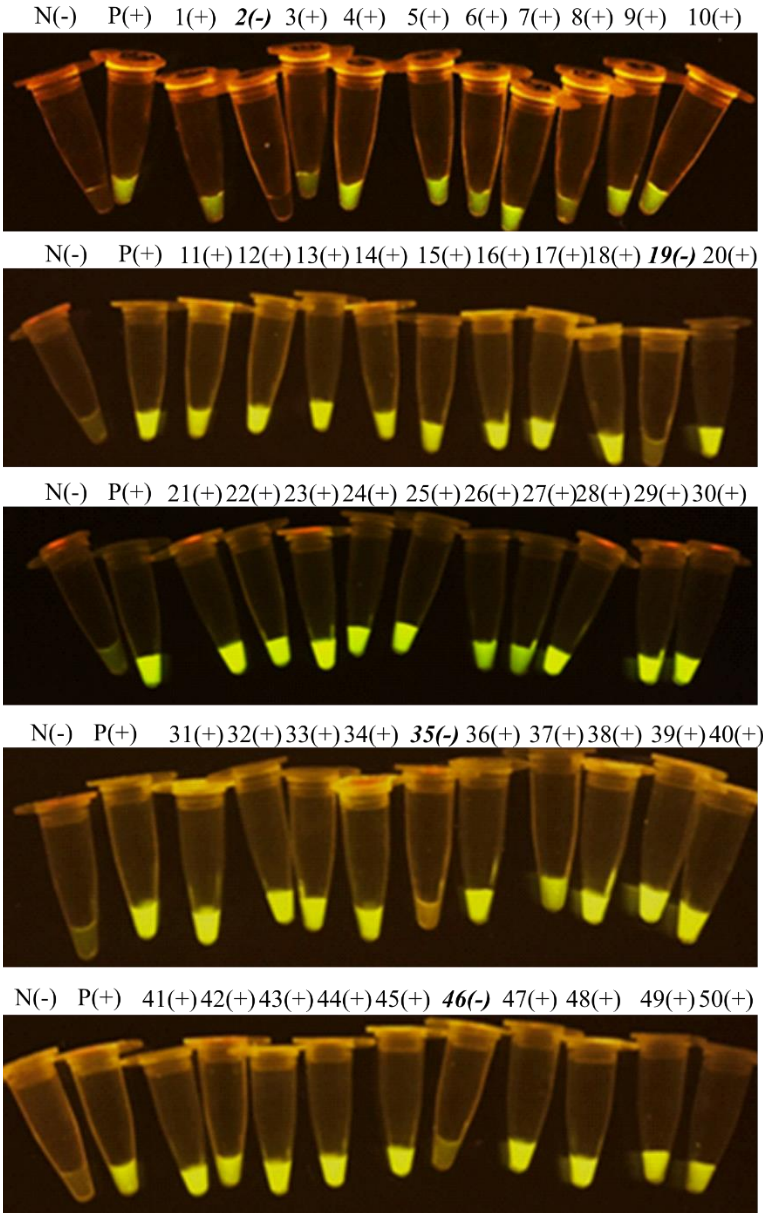

Supplementary Fig. 3: Images of RPA-CRISPR/CAS12a test results of genomic DNA extracted from blood samples of bovines infected with *A. marginale.* N: no template control, P: *msp4* recombinant plasmid DNA, (-) and (+) signify negative and positive for *A. marginale* infection, respectively.

2. Supplementary Table

RPA reaction

DNA (ng/µL)	DNA (copy/µL)	% Hit rate
3E-03 (3 pg)	400000	100
3E-04 (300 fg)	40000	100
3E-05 (30 fg)	4000	100
3E-06 (3 fg)	400	100
3E-07 (300 ag)	40	100
3E-08 (30 ag)	4	77.77
3E-09 (3 ag)	0.4	44.44

(A) 57

DNA (ng/µl)	Set 1	Set 2	Set 3	Set 4	Set 5	Set 6	Set 7	Set 8	Set 9
3E-03	+	+	+	+	+	+	+	+	+
3E-04	+	+	+	+	+	+	+	+	+
3E-05	+	+	+	+	+	+	+	+	+
3E-06	+	+	+	+	+	+	+	+	+
3E-07	+	+	+	+	+	+	+	+	+
3E-08	+	+	+	+	+	-	+	+	-
3E-09	-	-	+	+	+	-	-	+	-

(B)

PCR reaction

DNA (ng/µL)	DNA (copy/µL)	% Hit rate
3E-03 (3 pg)	400000	100
3E-04 (300 fg)	40000	100
3E-05 (30 fg)	4000	100
3E-06 (3 fg)	400	100
3E-07 (300 ag)	40	80
3E-08 (30 ag)	4	80
3E-09 (3 ag)	0.4	40

(C)

DNA (ng/µl)		Set 1	Set 2	Set 3	Set 4	Set 5
3E-03		+	+	+	+	+
3E-04		+	+	+	+	+
3E-05		+	+	+	+	+
3E-06		+	+	+	+	+
3E-07		+	+	-	+	+
3E-08		+	+	-	+	+
3E-09		-	-	-	+	+

(D)

Supplementary Table 1.

The starting DNA concentrations ranging from 3 pg/µl to 3 ag/µl were used in both RPA and PCR reaction with the same set of primers (F1F2R1). + represents detectable amplified product and - represents no detectable amplified product.

Sample#	PCR	RPA-CRISPR/Cas12a	Sample#	PCR	RPA-CRISPR/Cas12a
**1.**	+	+	**26.**	+	+
**2.**	+	-	**27.**	+	+
**3.**	+	+	**28.**	+	+
**4.**	+	+	**29.**	+	+
**5.**	+	+	**30.**	+	+
**6.**	+	+	**31.**	+	+
**7.**	+	+	**32.**	+	+
**8.**	+	+	**33.**	+	+
**9.**	+	+	**34.**	+	+
**10.**	+	+	**35.**	+	-
**11.**	+	+	**36.**	+	+
**12.**	+	+	**37.**	+	+
**13.**	+	+	**38.**	+	+
**14.**	+	+	**39.**	+	+
**15.**	+	+	**40.**	+	+
**16.**	+	+	**41.**	+	+
**17.**	+	+	**42.**	+	+
**18.**	+	+	**43.**	+	+
**19.**	+	-	**44.**	+	+
**20.**	+	+	**45.**	+	+
**21.**	+	+	**46.**	+	-
**22.**	+	+	**47.**	+	+
**23.**	+	+	**48.**	+	+
**24.**	+	+	**49.**	+	+
**25.**	+	+	**50.**	+	+

Supplementary Table 2:

Comparison of PCR-based and RPA-CRISPR/Cas12a detection of *A. marginale* infection using genomic DNA extracted from bovine blood samples.

(+) positive for *A. marginale* infection

(-) negative for *A. marginale* infection

Supplementary Table 3

Component of PCR, RPA and CRISPR/Cas12a reaction

1. PCR reaction for detection of *A. marginale* infection using genomic DNA extracted from bovine blood as templates.

PCR reaction component	Final concentration	Volume (µl)
5X Phusion HF buffer	1X	4
10 mM dNTPs	200 µM each	0.4
10 µM Forward primer	1 µM	1
10 µM Reverse primer	1 µM	1
DNA sample	50 ng	Variable
2 U/µl Phusion DNA polymerase	0.02 U/µl	0.2
Water	-	To 20 µl
		25 cycles

2. PCR reaction for LOD detection using *msp4* plasmid DNA as template.

PCR reaction component	Final concentration	Volume (µl)
10X buffer	1X	2.5
2 mM dNTPs	200 µM each	2.5
25 mM $MgSO_4$	1 mM	1
15 µM Forward primer 1 (F1)	0.3 µM	0.5
15 µM Forward primer 2 (F2)	0.3 µM	0.5
15 µM Reverse primer 1 (R1)	1 µM	1
DNA sample	Variable	1
1 U/µl KOD DNA polymerase	0.02 U/µl	To 25 µl
Water	-	
		25 cycles

Note: annealing temperature is 50ºC

3. RPA reaction

RPA reaction component	Final concentration	Volume (µl)
Rehydration buffer	1X	29.5
15 µM Forward primer 1 (F1)	0.3 µM	1
15 µM Forward primer 2 (F2)	0.3 µM	1
15 µM Reverse primer 1 (R1)	0.6 µM	2
DNA sample	-	1
Magnesium acetate (MgOAc)	14 mM	2.5
Water	-	To 50 µl

4. CRISPR/Cas12a reaction

CRISPR/Cas12a reaction component	Final concentration	Volume (µl)
10X NEBuffer 2.0	1X	1.5
300 nM crRNA	30 nM	1.5
750 nM Cas12a enzyme	50 nM	1
45 µM Fluorescent probe	1.5 µM	0.5
RPA product	-	1
Water	-	To 15 µl

Supplementary method

Full protocol for DNA extraction from whole blood samples

The DNA was extracted from the whole blood samples using a high-quality commercial kit (High Pure PCR Template Preparation Kit, ROCHE). Briefly, equal volume of blood sample and binding buffer (200 µl; 6 M guanidine-HCl, 10 mM urea, 10 mM Tris-HCl, 20% Triton X-100 (v/v), pH 4.4) were mixed together. Proteinase K was added to digest the contaminated proteins and incubated at 70ºC for 10 min. 100 µl of isopropanol was then added for DNA precipitation. The samples were transferred to purification columns and centrifuged at 8,000g for 1 min. After discarded the flow through liquid, 500 µl of inhibitor removal buffer (5 M guanidine-HCl, 20 mM Tris-HCl, pH 6.6) was added to the upper reservoir of the filter tube and centrifuged at 8,000g for 1 min. 500 µl of wash buffer (20 mM NaCl, 2 mM Tris-HCl, pH 7.5, 80% ethanol) was applied twice. The empty filter tube was lastly centrifuged to remove the residual wash buffer. To elute the DNA, the filter tube was transferred to a clean, sterile 1.5

ml microcentrifuge tube, where 50 µl of prewarmed elution buffer (10 mM Tris-HCl, pH 8.5) at 70ºC was added and centrifuged at 8,000g for 1 min. The NanoDrop 2000™ spectrophotometer (Thermo Scientific) was used to measure concentrations of purified DNA. The purity of extracted DNA was determined by A_{260}/A_{280} ratio. The optimum value for pure DNA is in the range of 1.7-2.0.
