# Supplementary material for: RPA-CRISPR/Cas12a assay for the diagnosis of bovine Anaplasma marginale infection
Source: Sci Rep. 2024 Apr 3;14:7820. doi: 10.1038/s41598-024-58169-6 (PMC10991388; doi:10.1038/s41598-024-58169-6)
Supplement: Supplementary file 2 — Supplementary Information 2. [file 41598_2024_58169_MOESM2_ESM.pdf]

Uncropped gel images and replicate results

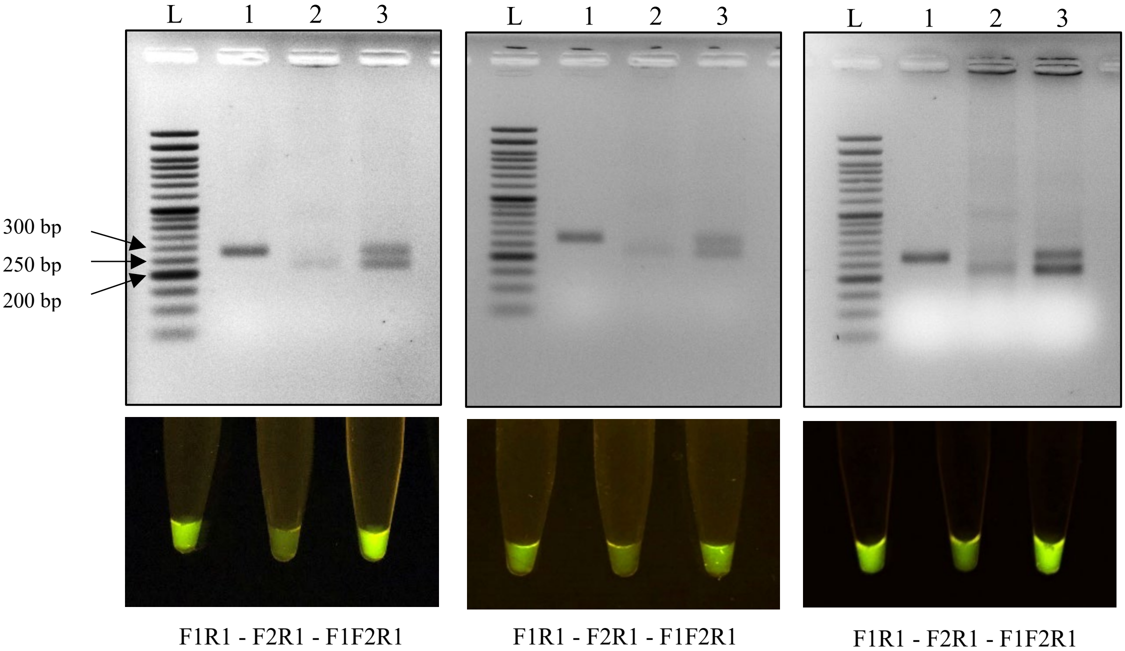

Figure 2B-C: Validation of RPA-CRISPR/Cas12a assay detection

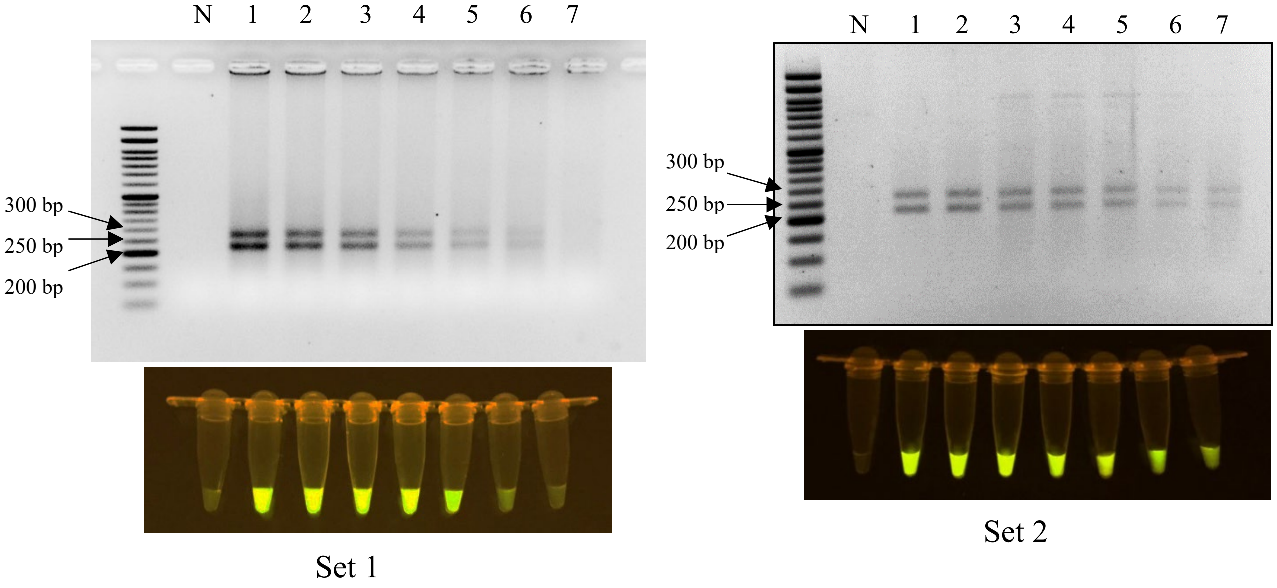

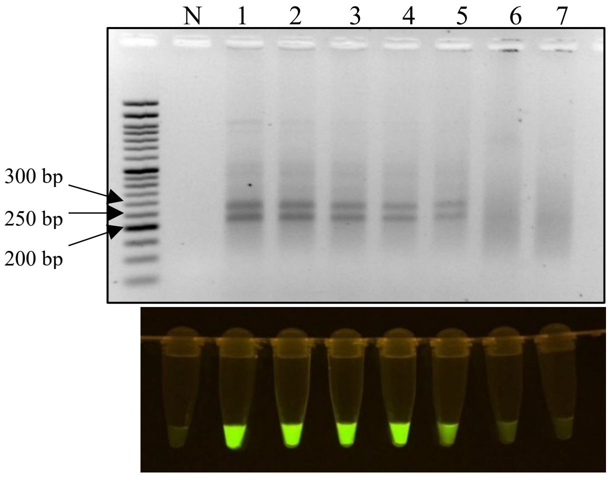

Set 3

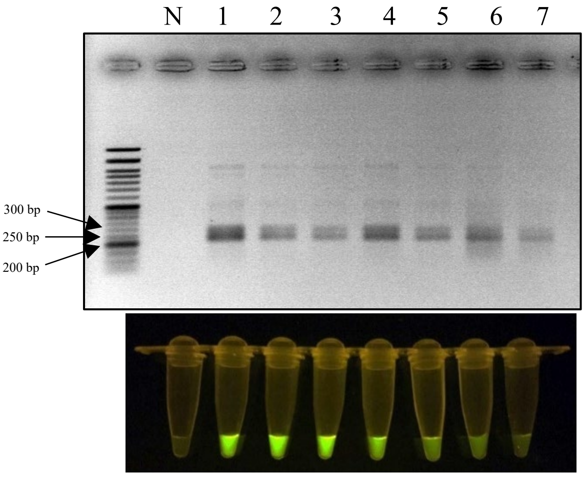

Set 4

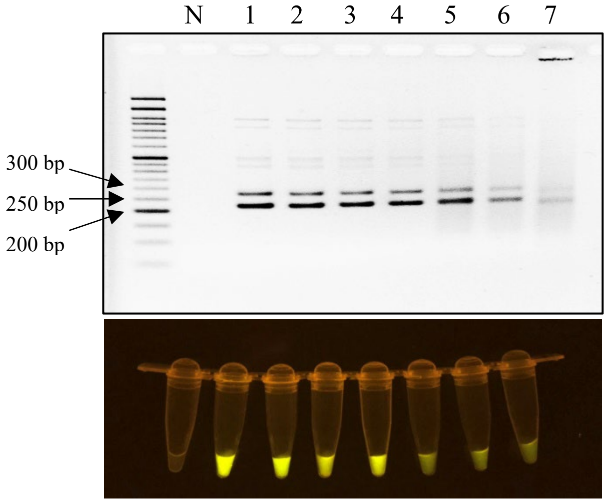

Set 5

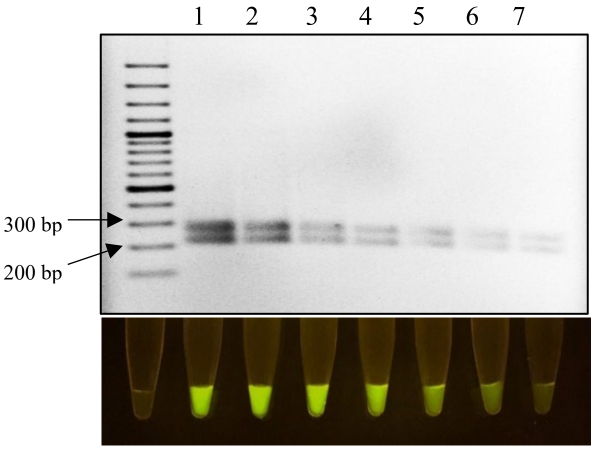

Set 6

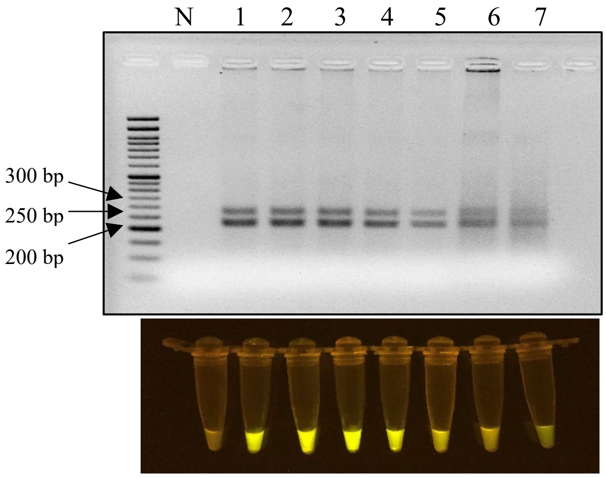

Set 7

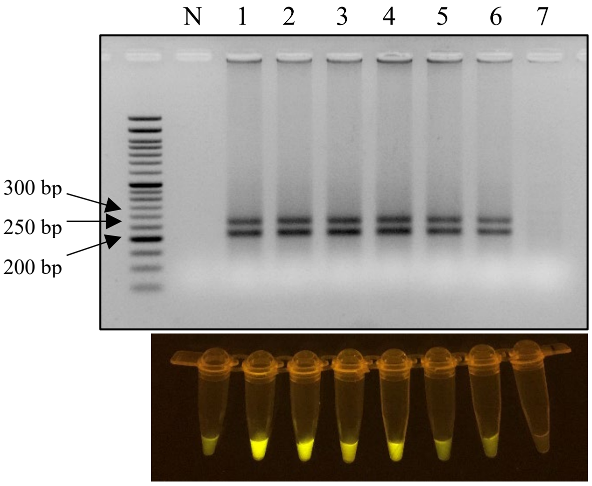

Set 8

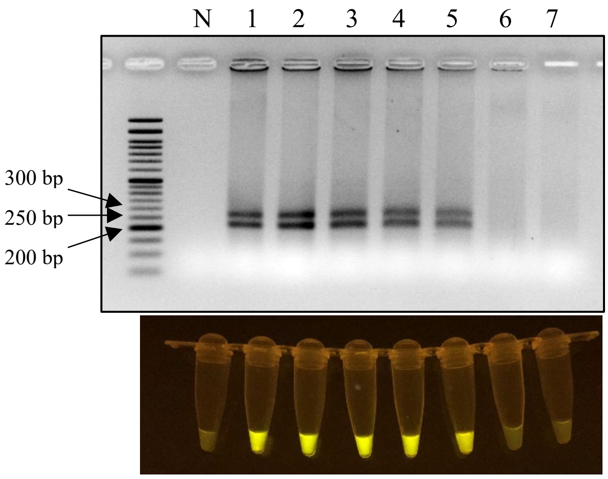

Set 9

Figure 2E-F: Validation of RPA-CRISPR/Cas12a assay detection

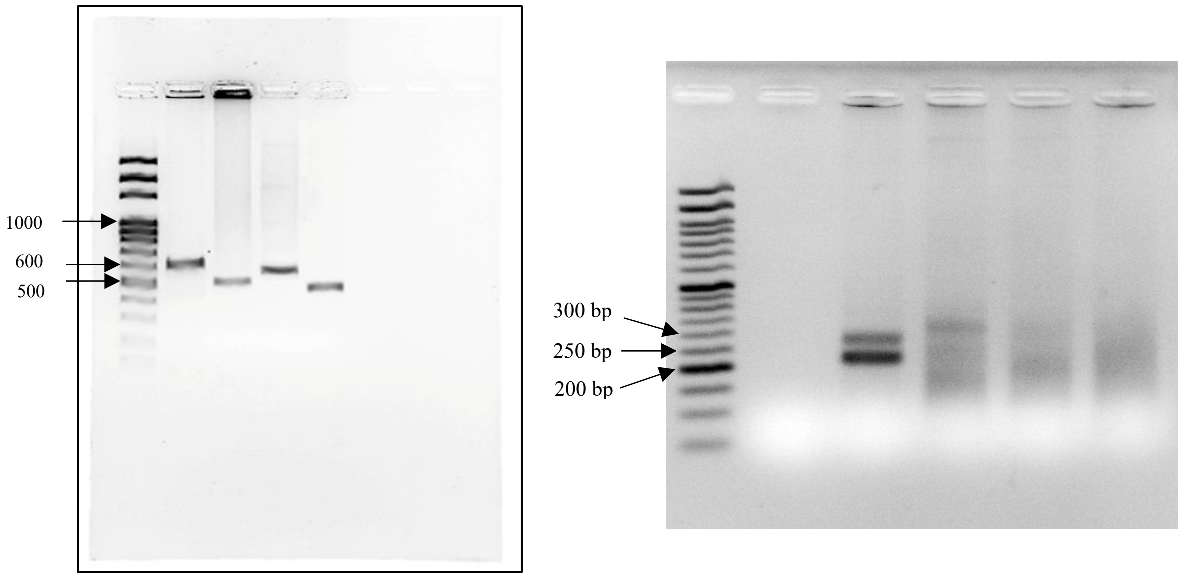

(A) (B)

Figure 3: Uncropped agarose gel images for figure 3A and 3B.

(3A): Lane 1: 100 bp marker (Solis BioDyne, Estonia) DNA ladder. Lane 2-5 are *msp5* of *A. marginale* (633 bp), *sbp* of *B. bovis* (584 bp), *p23* of *T. orientalis* (601 bp) and *18S* of *T. evansi* (538 bp), respectively.

(3B): Lane 1: ladder. Lane 2-5 are the samples of RPA products using primers for detecting *msp4* of *A. marginale*. The genomic DNA extracted from cattle blood infected with *A. marginale*, *B. bovis*, *T. orientalis*, or *T. evansi*, respectively were used as template in RPA reaction.

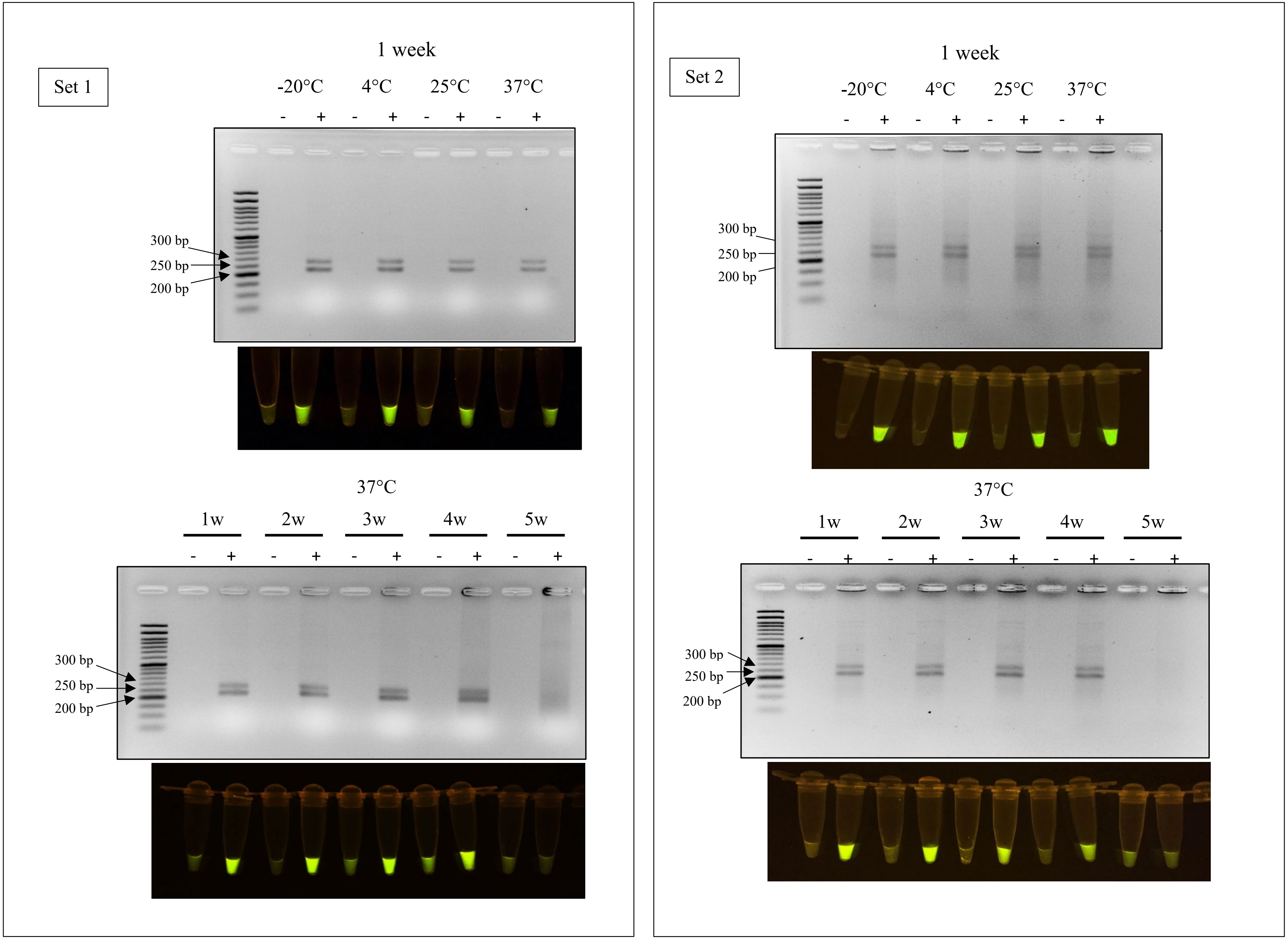

Set 1
1 week
-20°C
4°C
25°C
37°C
- + - + - + - +
300 bp
250 bp
200 bp
37°C
1w
2w
3w
4w
5w
- + - + - + - + - +
300 bp
250 bp
200 bp
Set 2
1 week
-20°C
4°C
25°C
37°C
- + - + - + - +
300 bp
250 bp
200 bp
37°C
1w
2w
3w
4w
5w
- + - + - + - + - +
300 bp
250 bp
200 bp

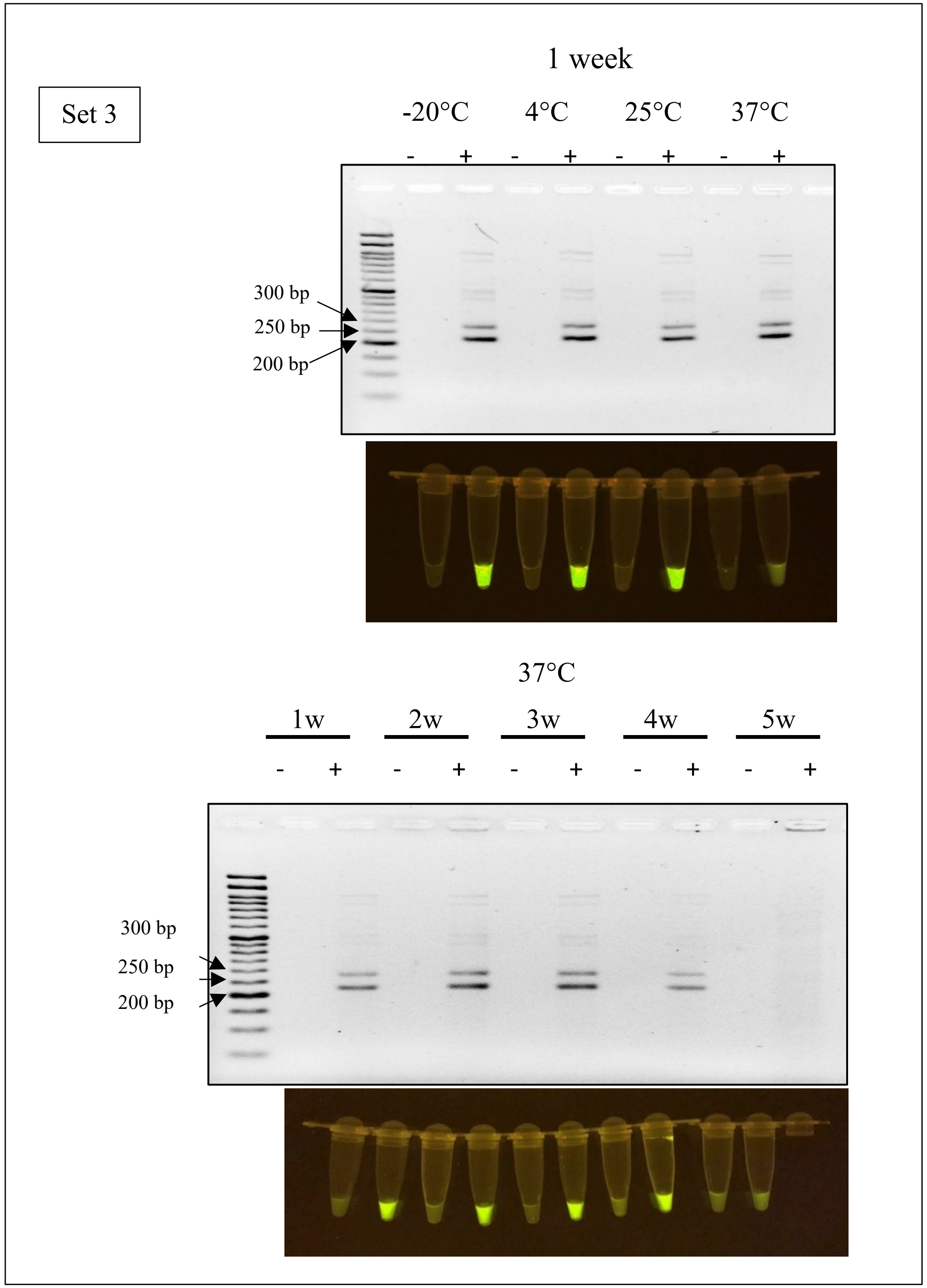

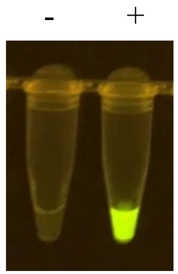

Figure 4: Uncropped agarose gel images and fluorescent signal of CRISPR/Cas12a reactions for figure 4A and 4B.

Figure 4

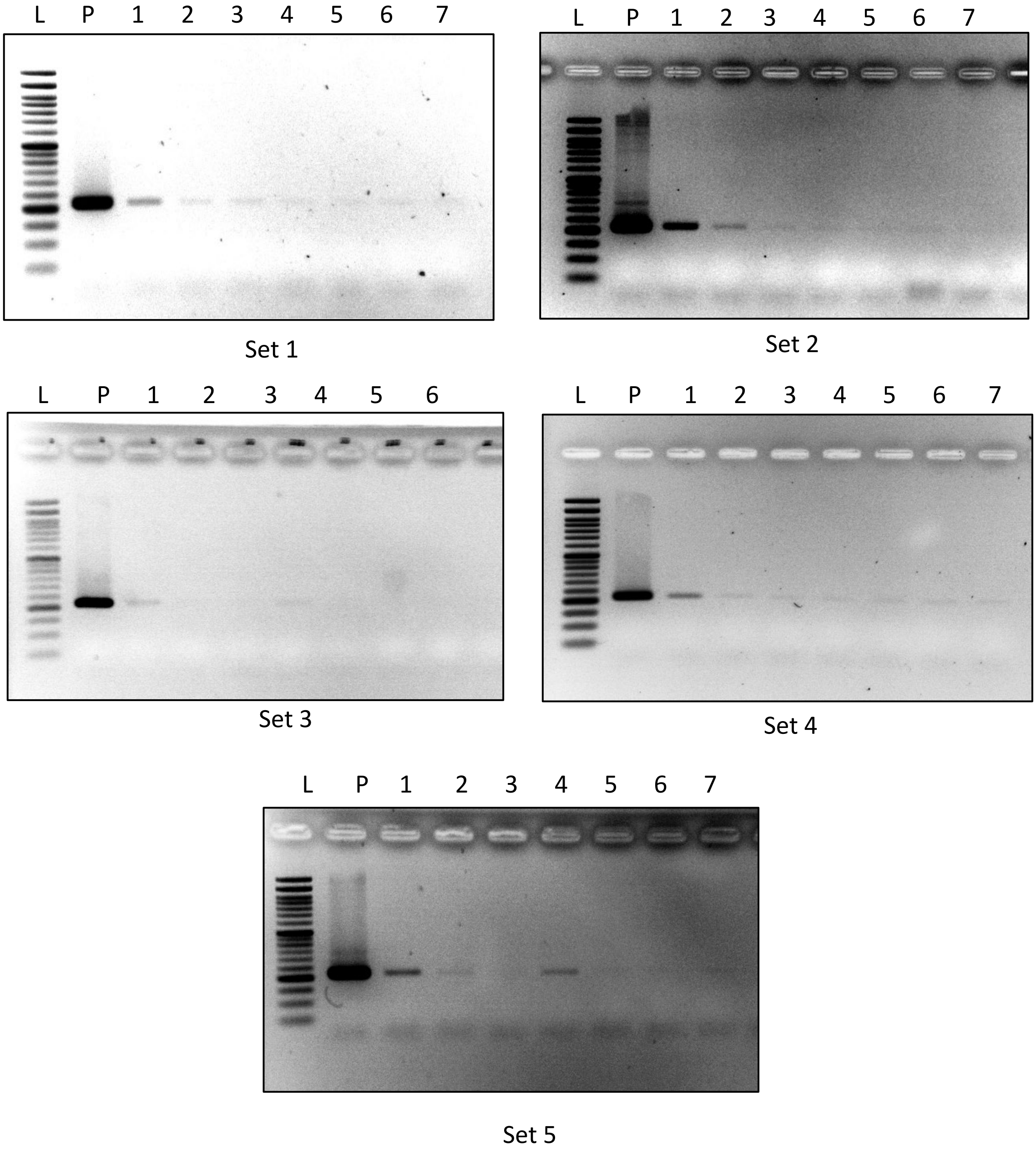

The LOD test of PCR reaction.

L is 50 bp DNA ladder (ExcelBand™), P is positive control where 3 ng/µl of plasmid DNA was used as template. Lane 1-7 are PCR product using various concentration of DNA template starting from 3 pg/µl, 300 fg/µl, 30 fg/µl, 3 fg/µl, 300 ag/µl, 30 ag/µl and 3 ag/µl, respectively.
